# Supplementary material for: Isolation of Peptide Inhibiting SGC-7901 Cell Proliferation from Aspongopus chinensis Dallas
Source: Int J Mol Sci. 2022 Oct 19;23(20):12535. doi: 10.3390/ijms232012535 (PMC9604521; doi:10.3390/ijms232012535)

[Sequence Analysis]

Start	: 2017-6-12 11:54:41
Reactor	: 1
Number of Cycles	: 25
Sequence Schedule	: C:\PPSQ30\SeqProg1\STGFD111.SCH
Sample Name	: 20170612 caomilan
Sample Amount(pmol)	: 25.0
Sample ID	:
Operator Name	:
Data File	: 20170612 caomilan
Start Number	: 1
Method File	: 20170612 caomilan.LCM
Batch File	: 20170612 caomilan.LCB
Data Folder Path	: C:\LabSolutions\Data\PPSQ30\20170612 caomilan
Number of Analyses	: 24 / 24
Standard File	: 20170405 PTH-AA_D01.lcd

[Sequence]

Glu	Lys	His	Tyr	Ala	Ala	Glu	Lys	Gly	Ile
Arg	Arg	Asp	Asp	Ile	Ile	His	Thr	Thr	Thr
Leu	Lys	Lys	Lys						

[Estimated Sequence]

	1	2	3	4	5	6	7	8	9	10
1st	Glu	Lys	His	Tyr	Ala	Ala	Glu	Lys	Gly	Ile
2nd	Gln	Tyr	Gly	Asp	Lys	Glu	Lys	His	His	Arg
3rd	Ala	Asp	Tyr	Phe	Ser	Lys	Ile	Gly	Ile	Thr
4th	Met	Trp	Asp	Asn	Asp	Leu	Gly	Gln	Asn	Leu
Reliability(%)	46.2	4.0	10.4	100.0	21.4	100.0	100.0	100.0	11.8	100.0

	11	12	13	14	15	16	17	18	19	20
1st	Arg	Arg	Asp	Asp	Ile	Ile	His	Thr	Thr	Thr
2nd	Asp	Asp	Pro	Pro	Tyr	His	Val	Gln	Gly	Gly
3rd	Asn	Ser	Ala	Tyr	Leu	Tyr	Ser	Ala	Phe	Leu
4th	Thr	Gln	Asn	Asn	Phe	Val	Thr	Tyr	Tyr	Val
Reliability(%)	100.0	100.0	100.0	100.0	100.0	52.2	11.4	12.1	100.0	11.6

	21	22	23	24
1st	Leu	Lys	Lys	Lys
2nd	Gly	Leu	Tyr	Asn
3rd	Phe	Gln	Asn	Pro
4th	Gln	Val	Val	Phe
Reliability(%)	42.7	14.9	100.0	100.0

[Evaluated Value]

	1	2	3	4	5	6	7	8	9	10
Asp	0.99	0.89	6.22	2.87	3.20	2.94	0.82	5.79	0.73	0.83
Glu	621.95	0.18	0.59	0.72	0.84	4.86	527.79	0.54	0.40	0.46
Asn	1.04	0.83	3.05	1.16	0.91	0.97	0.85	2.64	2.46	0.90
Gln	97.94	0.61	0.61	0.55	0.76	0.97	0.72	8.80	0.77	0.66
Ser	9.32	0.45	0.75	0.86	3.40	0.42	0.91	1.47	1.60	0.69
Thr	8.11	0.49	0.52	0.66	1.00	1.13	0.95	2.87	0.78	1.11
His	6.75	0.82	905.27	0.20	0.28	0.50	0.95	19.09	583.31	0.40
Gly	28.06	0.67	899.23	0.20	0.24	0.47	2.02	16.87	665.07	0.36
Ala	91.97	0.50	0.58	0.65	11.77	1257.20	0.27	0.28	0.37	0.55
Tyr	1.44	0.93	9.66	740.74	0.32	0.24	0.30	0.74	0.76	0.65
Arg	0.00	0.20	0.26	0.40	0.69	0.58	0.46	2.62	1.52	3.48
Met	90.58	0.20	0.25	0.42	0.48	1.72	0.62	0.00	0.00	0.00
Val	16.59	0.55	0.70	0.68	0.93	1.63	0.81	3.89	0.79	0.88
Pro	21.65	0.41	0.87	0.73	0.92	0.80	0.49	4.13	0.59	0.49
Trp	1.11	0.89	0.00	0.00	0.00	2.31	0.00	0.00	0.00	0.00
Phe	11.95	0.00	1.66	1.31	0.00	0.96	0.95	0.00	0.00	0.00
Lys	0.89	0.95	0.99	0.72	4.11	4.32	16.62	1342.99	0.29	0.31
Ile	10.82	0.51	0.71	0.71	2.12	0.27	8.17	5.32	11.63	533.60
Leu	24.35	0.44	0.80	0.73	2.66	3.39	0.91	4.79	0.84	0.98

	11	12	13	14	15	16	17	18	19	20
Asp	4.71	7.61	228.75	408.89	0.71	0.61	0.61	0.70	0.71	0.66
Glu	0.64	0.69	0.78	0.76	0.75	0.80	0.77	0.88	0.78	0.86
Asn	2.34	1.95	2.77	1.33	0.97	0.95	0.94	2.02	0.98	1.00
Gln	0.91	1.99	0.97	0.93	0.90	0.93	0.99	2.43	0.93	0.96
Ser	1.37	4.07	1.05	0.91	0.97	0.90	1.62	1.74	0.94	0.99
Thr	1.71	0.91	0.93	0.94	0.98	0.92	1.61	4.94	94.90	94.31
His	0.33	0.43	0.58	0.72	0.81	43.50	3.05	0.75	0.52	0.67

Gly	0.33	0.42	0.71	0.79	0.93	0.97	0.90	1.37	7.05	84.51
Ala	0.81	0.89	4.59	0.84	0.78	0.81	0.93	2.24	1.06	0.84
Tyr	0.78	0.87	1.16	2.20	3.00	1.52	0.98	2.18	1.12	1.57
Arg	90.98	77.31	0.64	0.54	0.57	0.71	0.82	0.96	0.94	0.95
Met	0.00	0.00	0.00	0.00	0.00	0.00	0.00	0.00	0.00	0.00
Val	0.92	0.86	0.97	1.24	0.91	1.13	1.68	1.72	1.05	1.99
Pro	0.00	0.00	5.43	4.50	0.69	0.49	0.00	0.00	0.00	0.00
Trp	0.00	0.00	0.00	0.00	0.00	0.00	0.00	0.00	0.00	0.00
Phe	1.13	1.00	0.00	0.99	1.50	0.99	0.98	0.98	1.25	0.90
Lys	0.43	0.74	0.98	0.91	0.68	0.99	0.86	1.98	0.96	0.99
Ile	0.48	0.33	0.42	0.79	250.05	207.54	0.63	0.59	0.59	0.65
Leu	1.41	0.97	2.44	0.91	2.52	0.97	0.94	1.04	1.12	4.90

	21	22	23	24
Asp	0.67	0.76	0.87	0.80
Glu	0.69	0.87	0.93	0.89
Asn	0.86	1.11	2.31	1.47
Gln	1.30	2.76	1.92	0.93
Ser	0.83	0.99	1.26	0.98
Thr	0.73	0.69	0.71	0.71
His	0.53	0.26	0.19	0.52
Gly	22.20	0.87	0.78	0.70
Ala	0.75	0.81	0.98	0.84
Tyr	0.91	0.99	2.41	0.98
Arg	0.92	1.26	2.08	0.96
Met	0.00	0.00	0.00	0.00
Val	0.99	2.08	2.15	0.97
Pro	0.00	1.58	1.13	1.17
Trp	0.00	0.00	0.00	0.00
Phe	1.50	0.97	1.39	1.04
Lys	1.07	153.06	147.76	197.63
Ile	0.69	0.48	0.88	0.89
Leu	86.19	107.80	0.91	0.76

[Amount Yield(pmol)]

	1	2	3	4	5	6	7	8	9	10
Asp	34.43	0.00	0.00	3.59	29.33	33.06	31.12	41.78	36.73	29.50
Glu	758.32	0.00	0.00	0.00	64.87	65.64	508.82	276.84	147.15	64.75
Asn	11.78	0.00	0.00	0.00	10.23	9.10	9.06	11.03	22.44	15.68
Gln	255.08	0.00	0.00	0.00	42.48	40.05	31.35	39.67	37.19	24.11
Ser	3.61	0.00	0.00	0.00	8.27	9.85	5.46	5.57	7.11	3.49
Thr	11.36	0.00	0.00	0.00	6.09	6.64	5.16	7.51	6.31	3.96
His	102.68	0.00	0.00	0.00	86.72	65.81	36.02	34.14	18.44	7.32
Gly	107.66	0.00	856.25	25.43	77.07	83.07	47.83	50.05	650.18	232.17
Ala	160.63	0.00	0.00	0.00	42.67	1037.09	280.84	80.92	32.61	15.64
Tyr	10.11	0.00	4.06	588.36	189.21	56.98	14.65	10.91	9.43	2.96
Arg	29.75	97.96	0.00	0.00	11.07	8.47	0.00	8.69	14.20	3.56
Met	128.01	0.00	0.00	2.37	0.00	4.28	0.00	0.00	2.56	0.00
Val	57.96	0.00	0.00	0.00	10.94	16.39	8.12	12.06	11.33	6.60
Pro	41.05	0.00	0.00	9.44	5.70	10.20	0.00	4.16	3.24	0.00
Trp	4.71	0.00	0.00	32.33	0.00	0.00	0.00	0.00	2.46	0.00
Phe	10.10	0.00	0.00	22.25	0.00	0.00	0.00	0.00	0.00	0.00
Lys	20.41	8.53	0.00	7.29	22.06	38.65	35.49	1223.89	407.83	0.00
Ile	43.10	0.00	0.00	9.62	20.49	48.14	16.53	0.00	0.00	759.13
Leu	47.51	0.00	0.00	13.77	15.42	24.78	10.09	20.95	18.13	15.20

	11	12	13	14	15	16	17	18	19	20
Asp	28.56	35.42	275.11	550.25	429.85	218.43	137.64	102.35	79.91	62.49
Glu	41.13	32.67	27.12	12.65	9.86	21.80	14.90	15.31	15.54	14.21
Asn	11.51	12.79	14.89	15.37	16.77	16.05	13.91	15.51	16.83	16.67
Gln	20.61	22.83	23.09	22.75	22.71	22.23	20.66	23.58	24.17	23.65
Ser	3.14	6.00	5.86	5.67	6.69	5.80	4.57	6.63	7.27	7.36
Thr	4.21	4.97	5.06	4.81	6.56	6.33	3.48	8.80	71.85	86.08
His	4.74	4.13	4.39	0.00	0.00	60.20	59.73	47.38	32.37	0.00
Gly	75.17	30.66	26.13	19.77	27.56	20.65	16.20	19.01	26.07	125.99
Ala	11.97	12.76	15.73	11.01	10.97	10.15	7.58	8.46	11.27	10.58
Tyr	1.61	3.46	3.62	2.76	5.72	7.53	5.54	5.96	10.49	12.60
Arg	96.36	101.50	68.46	34.37	18.01	17.58	9.37	8.93	10.70	17.01
Met	0.00	0.00	0.00	0.00	0.00	0.00	0.00	0.00	0.00	0.00
Val	5.93	5.54	5.25	5.77	4.91	5.10	5.43	6.29	6.89	7.58
Pro	0.00	0.00	1.62	5.10	3.13	0.00	0.00	0.00	0.00	0.00
Trp	0.00	0.00	0.00	0.00	0.00	0.00	0.00	0.00	0.00	0.00
Phe	0.00	0.00	0.00	0.00	0.00	0.00	0.00	0.00	0.00	4.20
Lys	0.00	0.00	38.52	33.17	0.00	0.00	0.00	0.00	42.32	50.72

Ile	361.02	156.56	52.91	38.97	366.50	365.65	239.17	151.84	76.25	58.46
Leu	15.88	16.38	20.32	15.28	17.73	17.38	15.92	11.71	16.96	23.14

	21	22	23	24
Asp	43.64	35.75	32.02	27.21
Glu	11.28	10.44	16.67	17.15
Asn	13.86	14.62	16.96	18.24
Gln	21.24	23.65	25.73	24.90
Ser	4.52	5.11	5.90	6.66
Thr	58.70	40.69	30.59	23.43
His	0.00	0.00	0.00	0.00
Gly	130.28	110.45	89.62	65.34
Ala	5.65	3.49	4.36	6.11
Tyr	7.53	6.13	7.91	10.80
Arg	9.13	7.92	8.10	11.26
Met	0.00	0.00	0.00	0.00
Val	5.53	7.00	7.77	8.42
Pro	0.00	0.00	0.00	0.00
Trp	0.00	0.00	0.00	0.00
Phe	0.00	0.00	0.00	4.01
Lys	45.92	157.87	235.79	313.12
Ile	38.78	25.13	0.00	0.00
Leu	113.70	147.12	132.66	104.68

[Percent Yield]
Amino Acid : Ala,Val,Leu
Initial Yield(%) : 1018.96
Repetitive Yield(%) : 95.67
Correlation Coef. : -0.238
Number of Data : 3

[Repetitive Yield(%)]
Ala : 2430.24(5- 6)

Initial Yield(%): 1018.96 Repetitive Yield(%): 95.67

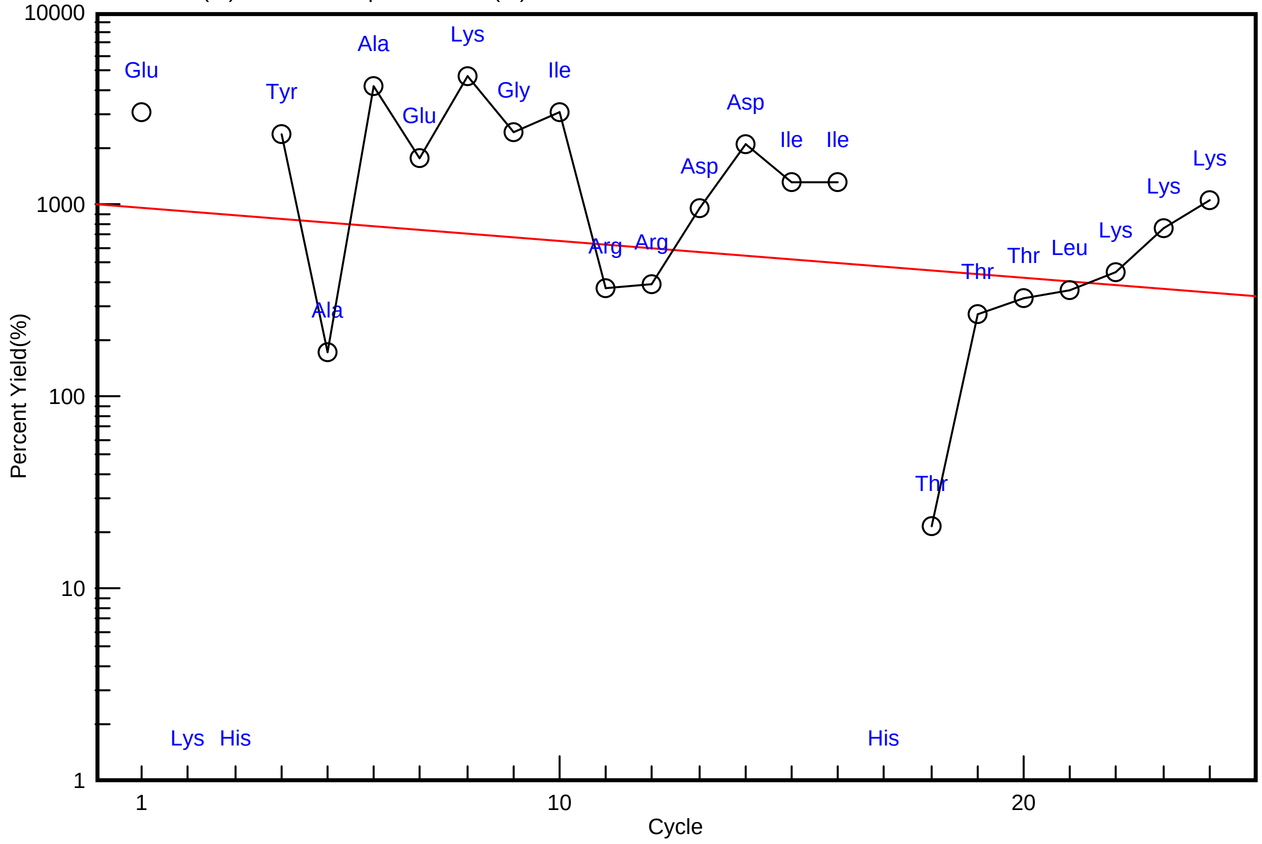

Supplement: Supplementary file 1 [file ijms-23-12535-s001.zip › PpsqDtpr - 20170612 caomilan.pdf]
