# Supplementary figures and images for: Isolation of Peptide Inhibiting SGC-7901 Cell Proliferation from Aspongopus chinensis Dallas
Source: Int J Mol Sci. 2022 Oct 19;23(20):12535. doi: 10.3390/ijms232012535 (PMC9604521; doi:10.3390/ijms232012535)

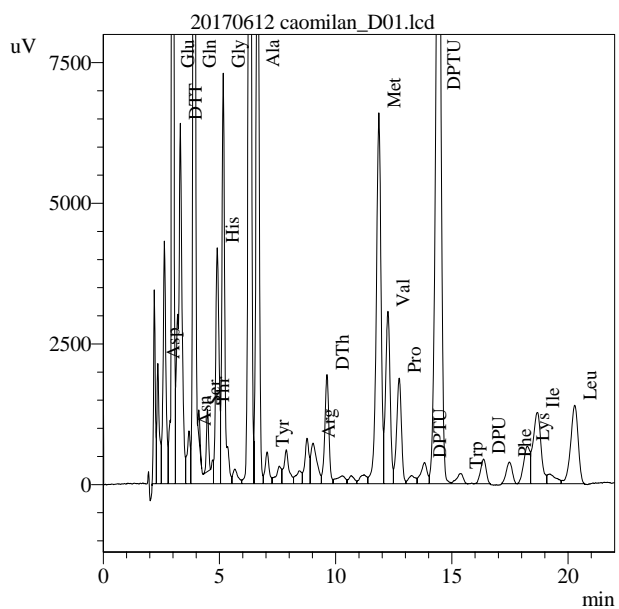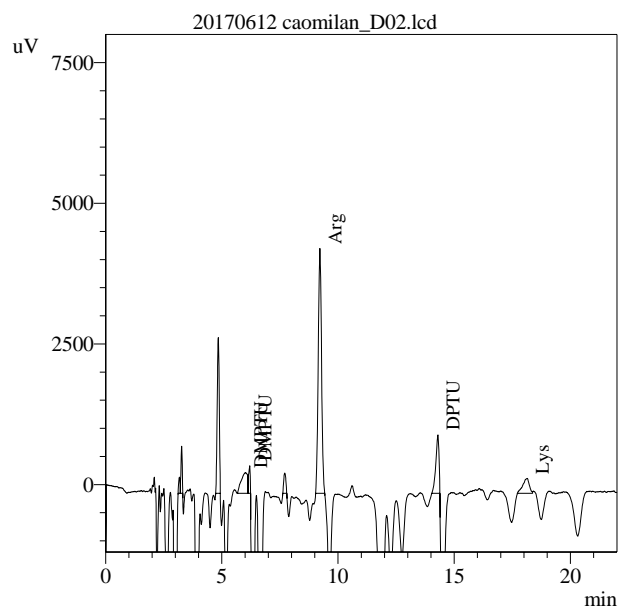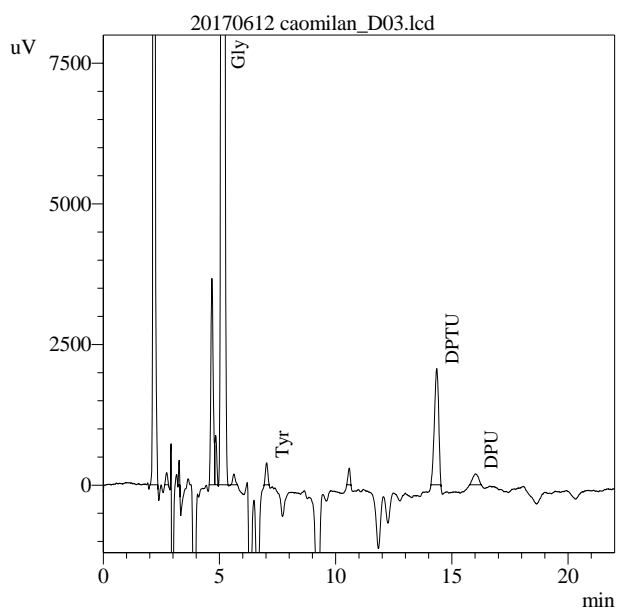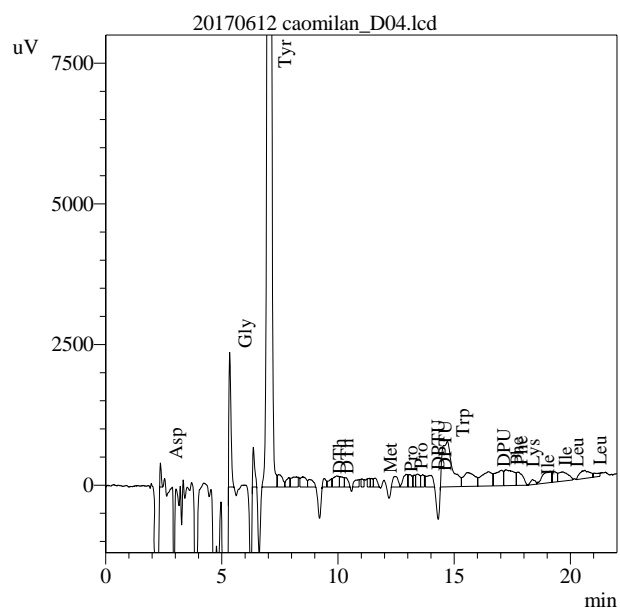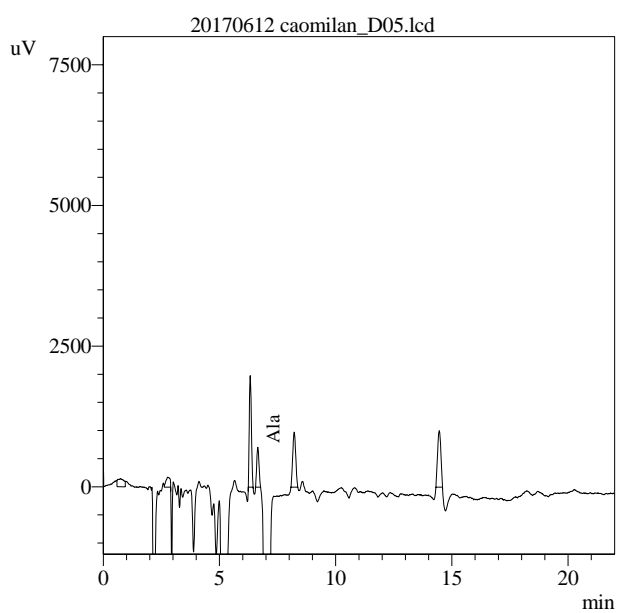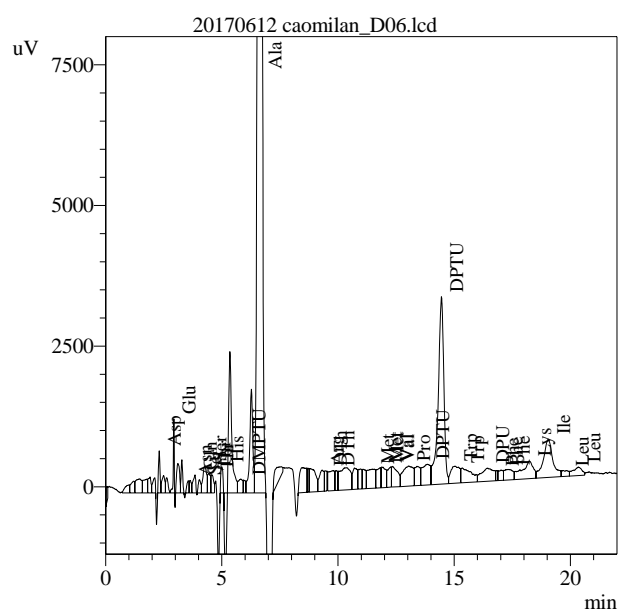

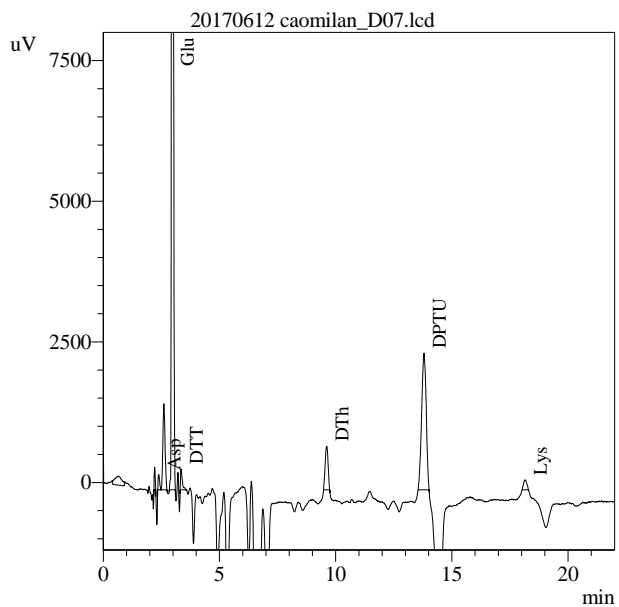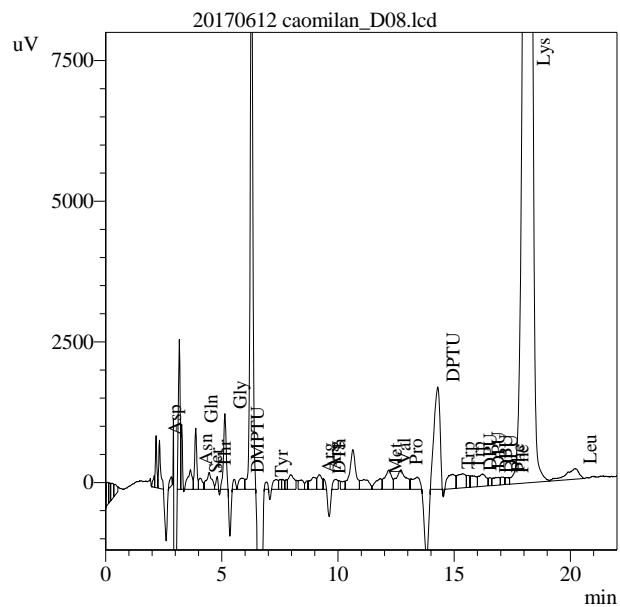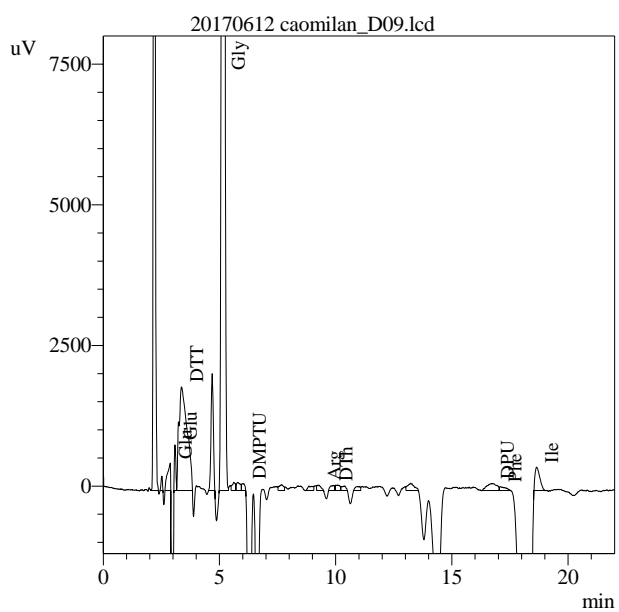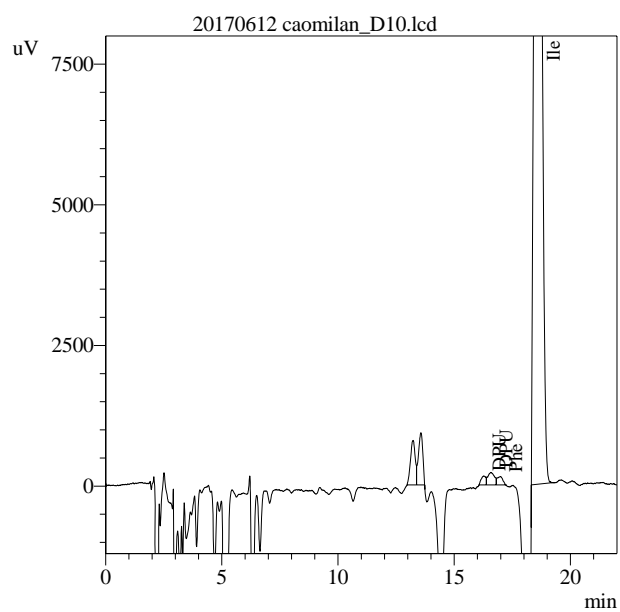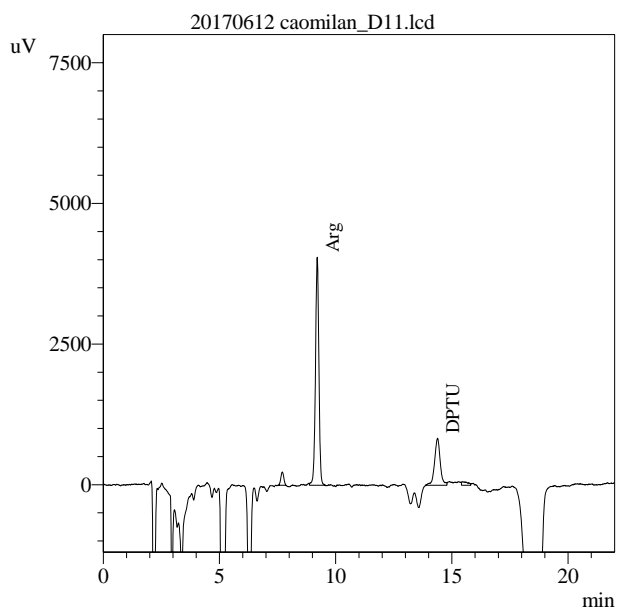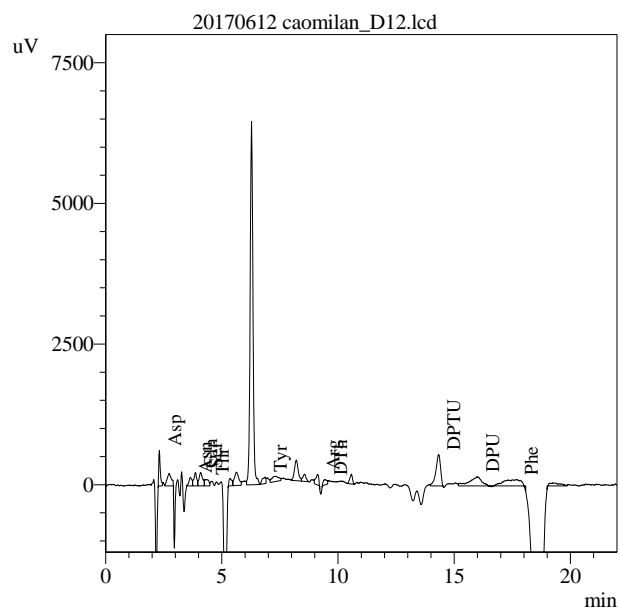

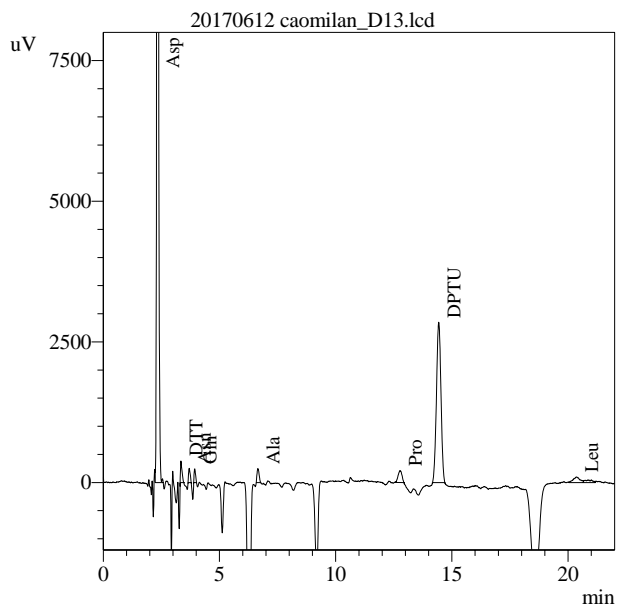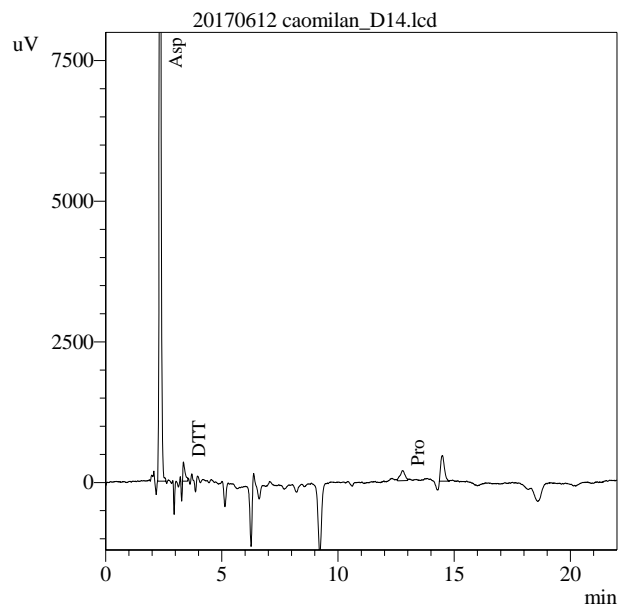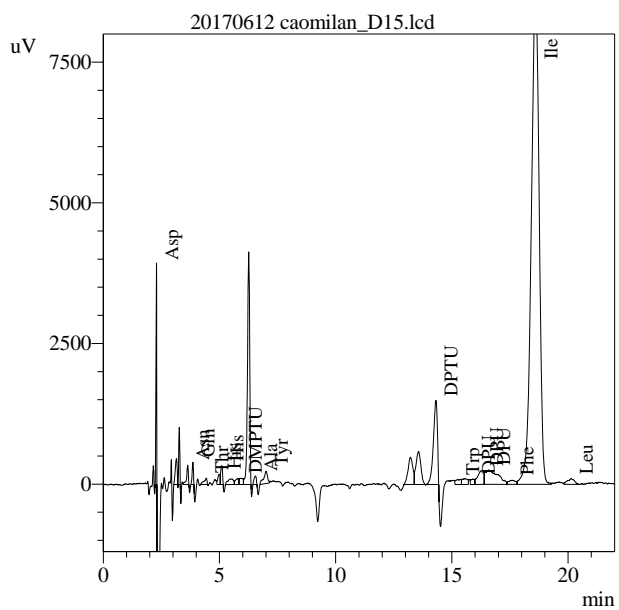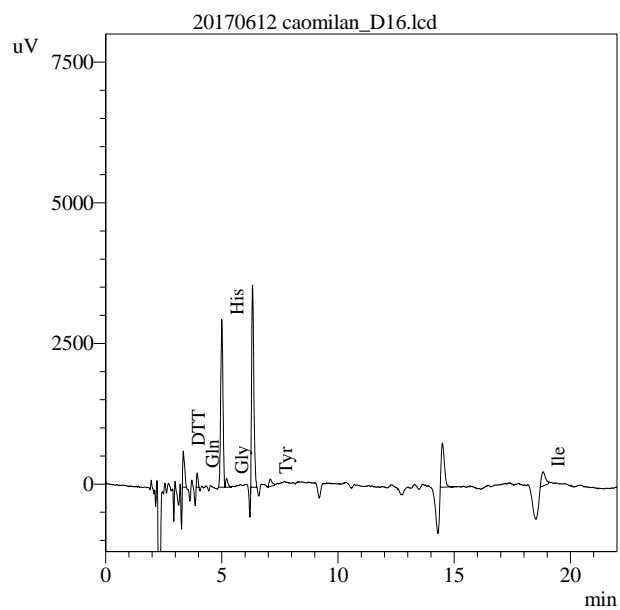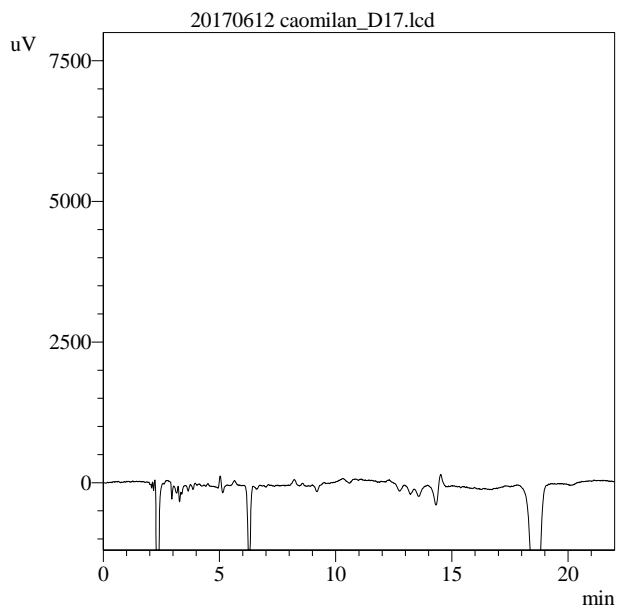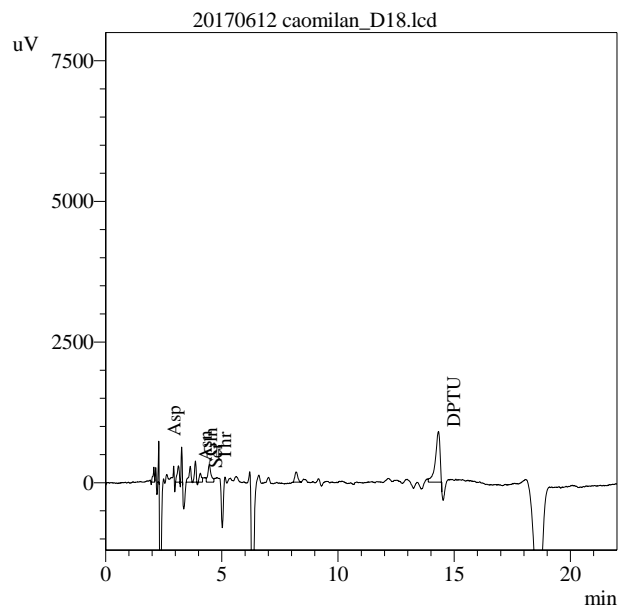

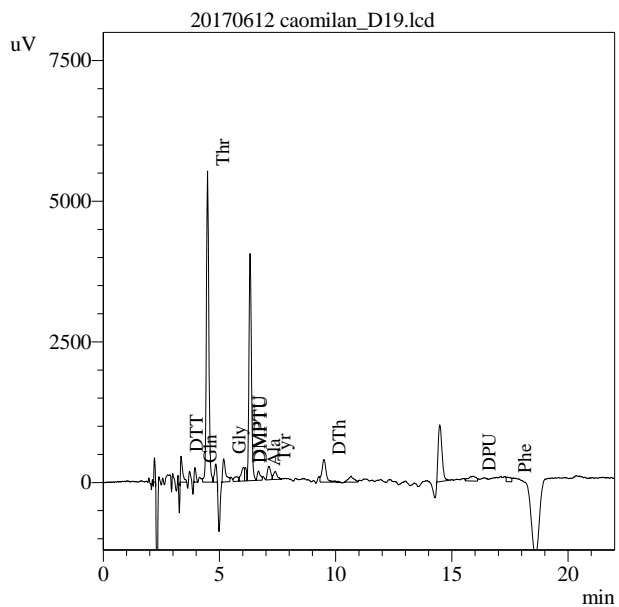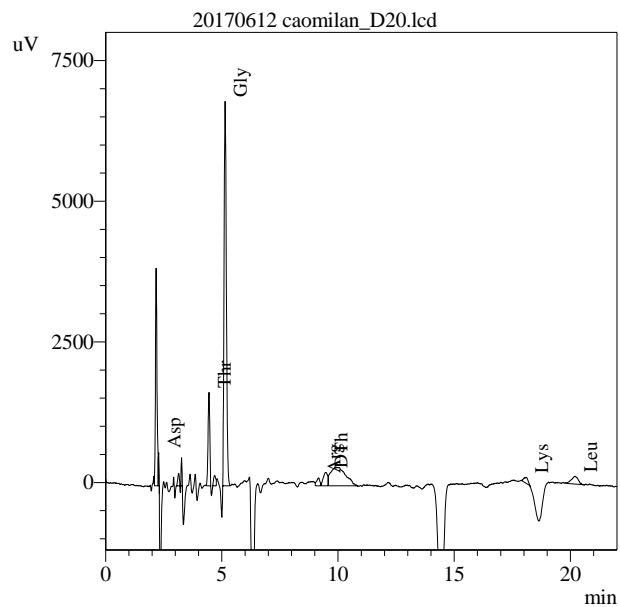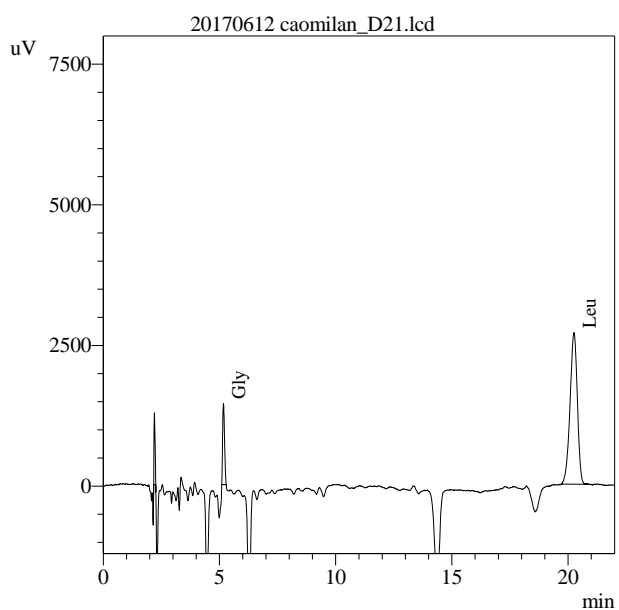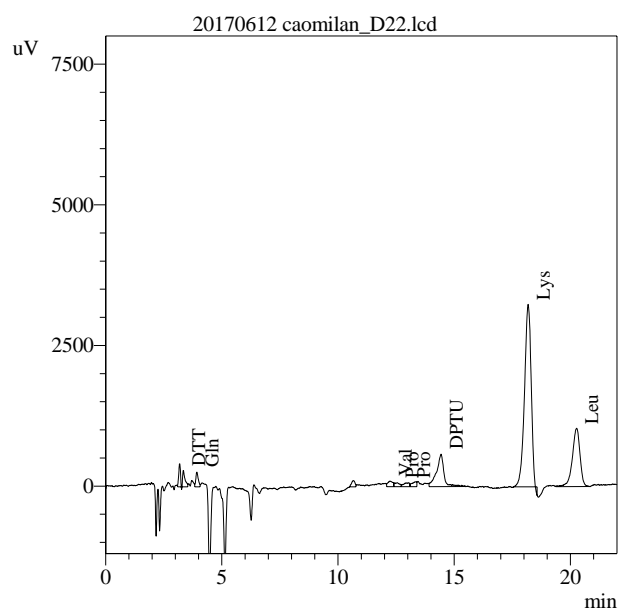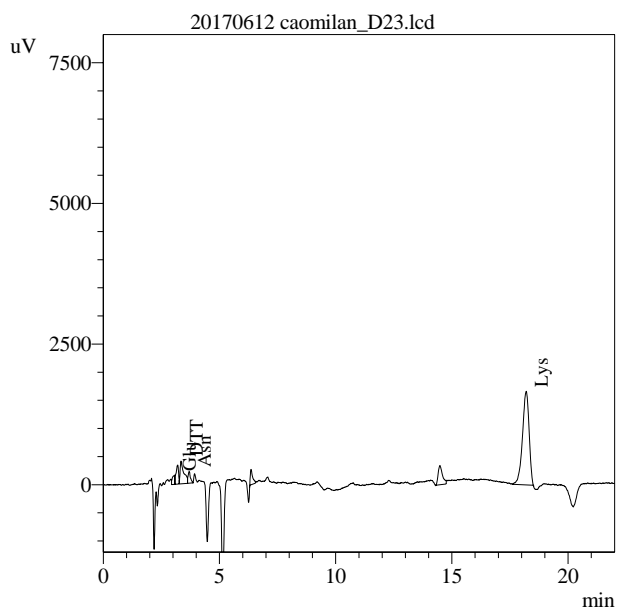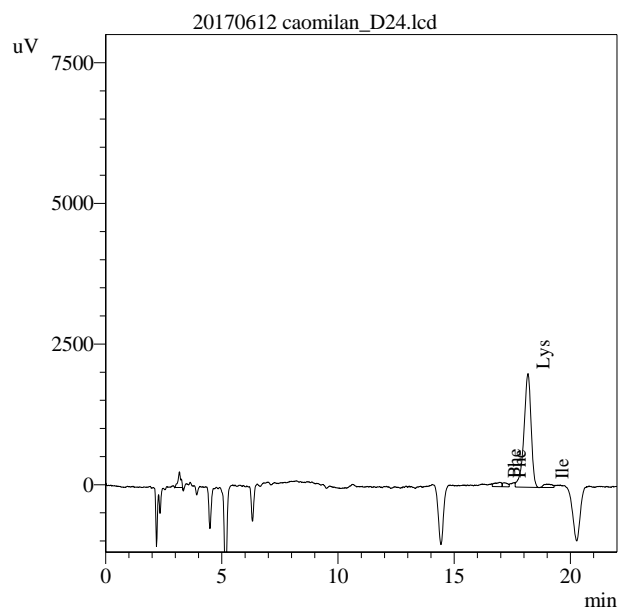

Supplement: Supplementary file 1 [file ijms-23-12535-s001.zip › PpsqSysReport_3_3_0.pdf]
